# Experimental investigation of the creep behaviour of remoulded loess under different levels of compactness

**DOI:** 10.1371/journal.pone.0262456

**Published:** 2022-01-24

**Authors:** Hao Tang, Jinzhi Luo, Zhao Duan, Dongpo Wang, Shengwen Qi

**Affiliations:** 1 Geological Research Institute for Coal Green Mining, Xi’an University of Science and Technology, Xi’an, China; 2 College of Geology and Environment, Xi’an University of Science and Technology, Xi’an, China; 3 State Key Laboratory of Geohazard Prevention and Geoenvironment Protection, Chengdu University of Technology, Chengdu, China; 4 Institute of Geology and Geophysics, Chinese Academy of Science, Beijing, China; Institute of Earth and Environment, Chinese Academy of Sciences, CHINA

## Abstract

Generally, a loess high fill project will undergo a time-dependent deformation and settlement process for a long time after the initial fill. Understanding the creeping behaviour of compacted loess is an important part of determining the stability of a compacted loess foundation. To study the creep behaviour of remoulded loess under different levels of compactness, we performed triaxial shear and triaxial creep tests using Q_2_ loess specimens obtained from the new district of Yan’an city. Based on laboratory test results, the triaxial shear and creep characteristics of remoulded loess under different levels compactness are summarised. The regularity of instantaneous strain, creep strain, total accumulated strain and initial shear modulus were analysed and the relationship between the compactness and long-term strength of remoulded loess is provided. It was found that the remoulded loess becomes harder and its long-term strength increases with an increase in compactness. Furthermore, we propose a new creep model (HD), based on the hardening-damage mechanism, and have derived one-dimensional (1D) and three-dimensional (3D) creep equations based on this new creep model. This new creep model is flexible enough to fit the typical creep test curves of remoulded loess, while perfectly describing the tertiary creep stage. Finally, the sensitivity of the HD creep model parameters was analysed; the results indicate that the parameters denoted as *α*, *γ*, and *β* significantly affect the morphological changes and various stage characteristics are represented by the creep curve.

## 1 Introduction

Loess is widely distributed all over the world, including North and South America and some areas of Europe, but is especially common throughout Asia. In northern China, there is a 440,000 square kilometre loess plateau [1]. When economic development occurs, some large-scale loess movement projects are required, such as excavating a mountain to build a city in Yan’an for the Yan’an city new district construction project [2]. Excavation, filling and compaction of loess will inevitably occur during such a huge construction project [3]; therefore, an in-depth understanding of the mechanical properties of compacted loess is very important for safety in loess engineering. Creep is an important mechanical property of the long-term deformation response of compacted loess. It is recognised that creep accumulation is related to the failure of many loess projects [4–6]. Likewise, understanding the creep properties of loess and determining its long-term strength is significant in the design and stability evaluation of loess engineering [7, 8].

Previous research has made some progress toward understanding the creep behaviour of loess. Yubov [9] investigated loess soil creep under complex stress conditions with varied water contents. Zhou et al. [10] studied the effects of confining pressure and temperature on the creep characteristics of frozen loess and established a rate-dependent constitutive model. Xie et al. [11] performed triaxial creep tests on loess-like soil and analysed the relationship between creep characteristics and microstructure. Tang et al. [12] conducted an experimental analysis of the creep characteristics of loess samples with different moisture contents and proposed a new two-element creep model. In addition, some scholars [13–15] have carried out creep tests on remoulded Q_3_ loess, including an analysis of the creep characteristics of loess under different levels of compactness and with various moisture contents, each establishing relevant models. Generally, the best way to describe the creep characteristics of geotechnical materials is to build a creep model. Proposing a creep model with a simple structure that better simulates the entire creep deformation process has become an objective for many researchers. Due to the pioneering work of Scott-Blair [16], who proposed a fractional element analogous to the classical Newtonian dashpot, many creep models based on fractional calculus have been developed in recent years. Rogers [17], Bagly and Torvik [18], and Koeller [19] have performed remarkable studies on creep models using fractional calculus. Welch et al. [20] proposed a four-parameter creep model to characterise the viscoelastic creep of polymeric materials using fractional calculus. Zhou et al. [21, 22] proposed a fractional derivative model to describe the creep behaviour of rock salt. Yin et al. [23] established a variable-order fractional creep model to describe the evolution of the mechanical properties of soft soils. It has been proven that fractional calculus is a good mathematical tool for constructing creep models, but there remain some defects in describing the tertiary creep of geotechnical materials. The third stage of accelerated creep involves nonlinear visco-elastic plasticity, so the third stage of the fractional creep model is not fully reflected. To solve this problem, a damage factor has usually been applied when building the model. Many scholars [24–27] have developed creep models for geotechnical materials by applying a damage factor; the results showed the advancement of the technology. Comprehensive, quantitative studies of the creep characteristics of remoulded Q_2_ loess still number relatively few and the effects of compactness are poorly understood. Therefore, this research has great significance for theoretical and engineering applications.

In the new district of Yan’an city, the long-term deformation of compacted loess is related to the stability of foundations. To understand the creep behaviour of compacted loess in this area, triaxial creep tests of remoulded loess with various levels of compactness were conducted. Furthermore, based on the principle of hardening damage, a new creep model (HD) was proposed using fractional calculus and damage mechanics. The new model was validated by fitting the test data of remoulded loess under different deviatoric stresses. The sensitivity of the HD creep model parameters was also analysed.

## 2 Materials and methods

### 2.1 Test specimens

**Fig 1:**
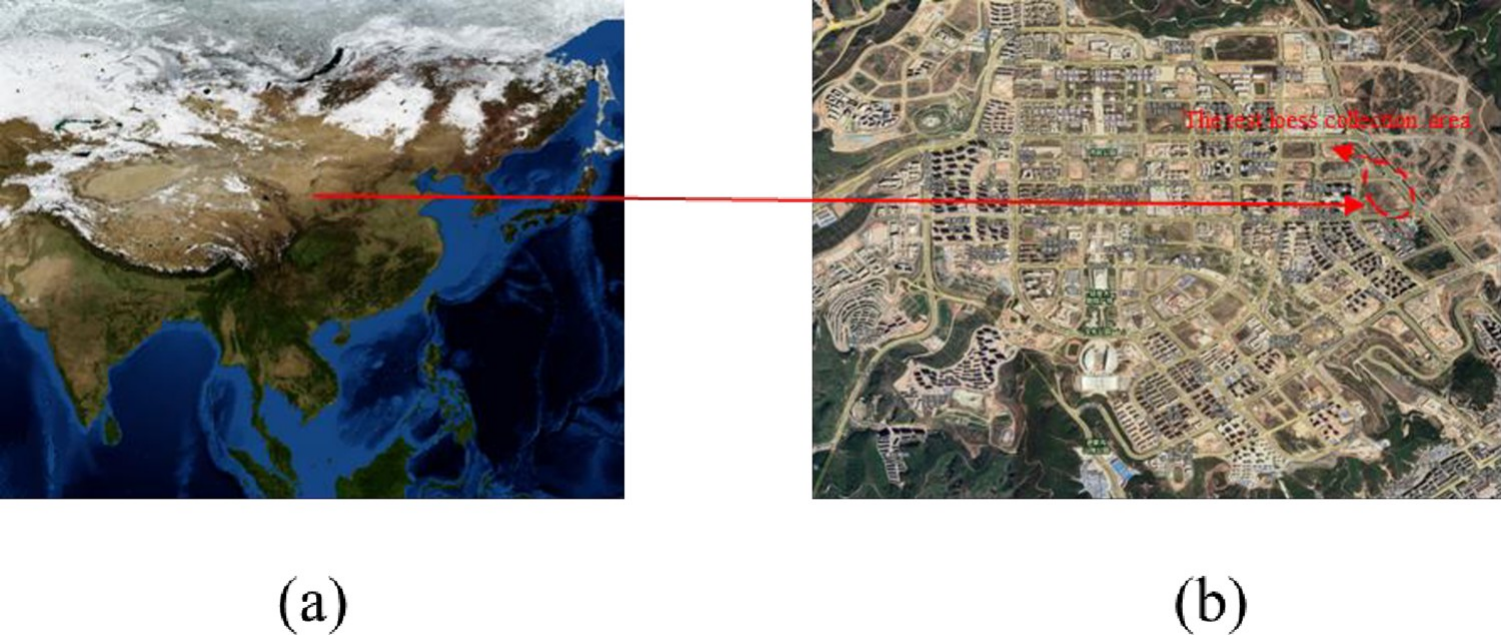
The loess sample collection area. ((a) and (b) are publicly available and free, the source is http://https://eol.jsc.nasa.gov/).

The loess material selected for the laboratory experiments was collected from the L6 loess layer in the new district of Yan’an city (Fig 1), which belongs to the quaternary middle Pleistocene loess, also known as Q_2_ loess. Through field investigation, the soil particles of the undisturbed loess were seen to be relatively uniform with a tawny colour and rich in vertical joints. The basic physical property parameters of the loess samples were determined following relevant ASTM standard test methods, as presented in Table 1.

**Table 1:** Physical property parameters of the loess samples.

ω* / %	*ω*_L_* / %	*ω*_P_* / %	*ω*_OP_* / %	ρ* / g/cm^3^	*ρ*_d_*/ g/cm^3^	*ρ*_dmax_*/ g/cm^3^	Cu*	Cc*
13.3	28.5	18.7	16.0	1.74	1.54	1.79	1.72	1.47

*ω: natural moisture content; *ω*_*L*_: liquid limit; *ω*_*P*_: plastic limit; *ω*_*OP*_: optimum moisture content; *ρ*: natural density; *ρ*_*d*_: dry density; *ρ*_*dmax*_: maximum dry density; *Cu*: non-uniformity coefficient; *Cc*: curvature coefficient.

**Fig 2:**
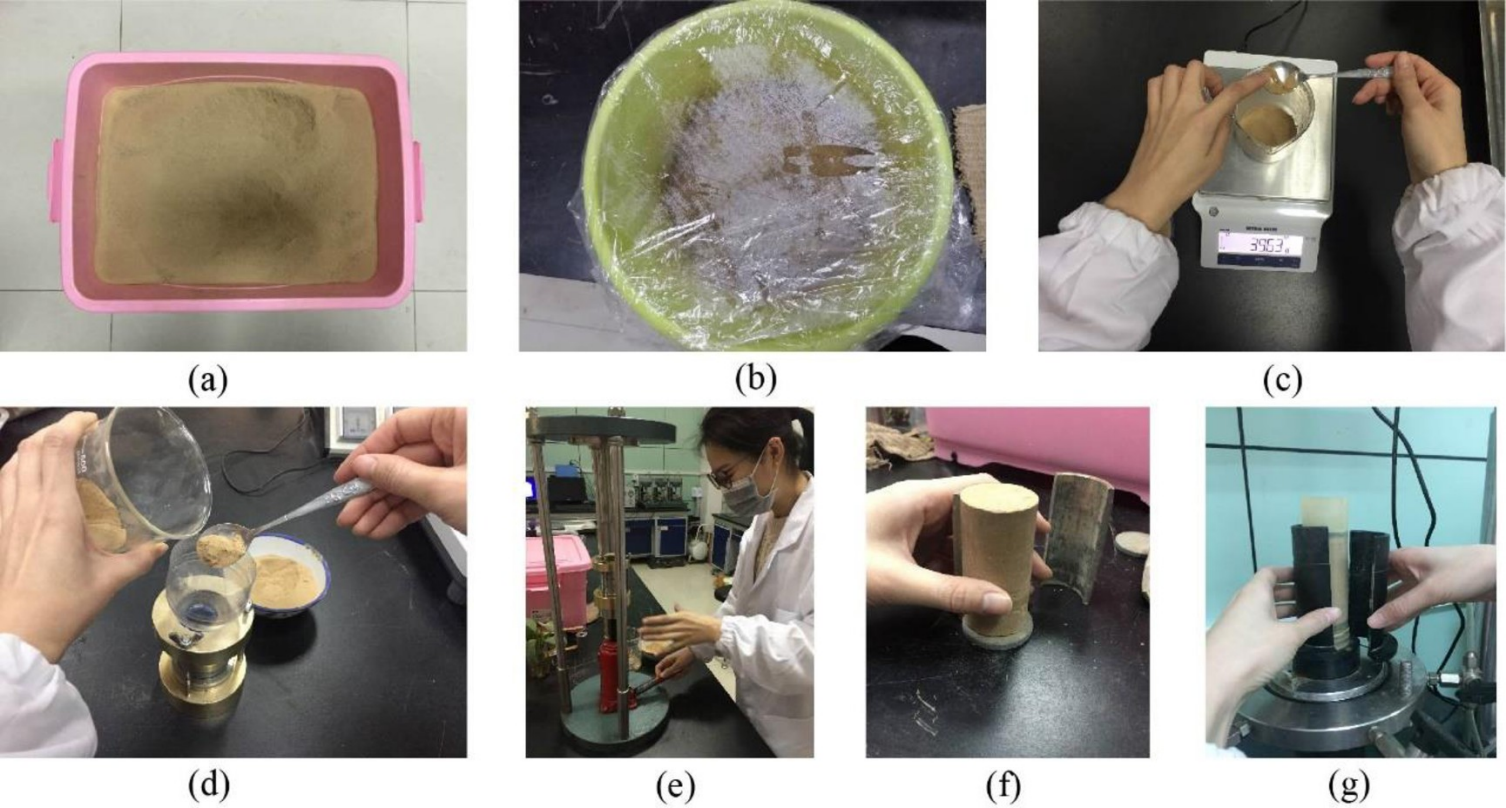
Specimen preparation. (a) Crushed and dried loess; (b) loess with a moisture content of 10%; (c) the amount of required loess is weighed; (d) transfer of loess into the sample; (d) specimens made by compaction; (f) specimen preparation completed; (g) specimens installed and prepared for testing.

The purpose of this study is to explore the creep characteristics of remoulded loess under different levels of compactness. First, as shown in Fig 2, the undisturbed loess was initially crushed and dried in an oven, then the crushed loess was sieved through a 2mm screen; second, the screened loess was mixed with water to a moisture content of 10%, then sealed in a basin for at least two days to achieve moisture homogenization; third, a balance was used to weigh the amount of soil required for cylindrical specimens with different compactness (0.84, 0.89, 0.95, and 0.99), then the weighed soil was placed into a cylindrical mould, and the remoulded specimens with a diameter and height of 39.8 and 80 mm, respectively, were made by means of static compaction. Finally, the specimens were kept in an incubator for follow-up testing.

### 2.2 Test procedures

**Fig 3:**
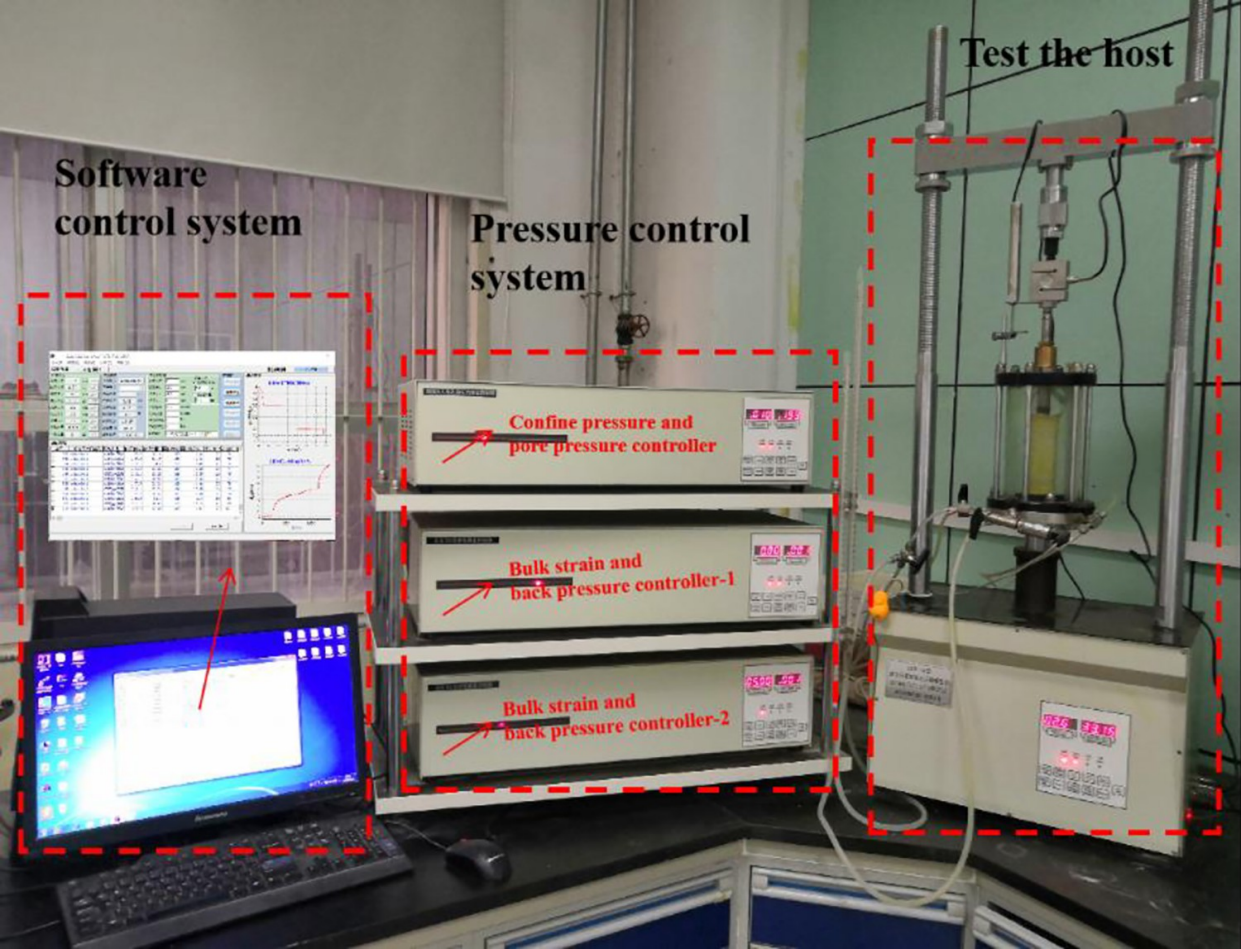
Triaxial creep shear apparatus on SLB-1A stress-strain controlled test machine.

The test equipment used in this study was the triaxial creep shear apparatus, SLB-1A stress-strain controlled test machine, as shown in Fig 3. The range value of the axial load was 0–20 kN with an accuracy of ±1% and the range value of the confining pressure was 0–1.99 MPa with an accuracy of ±0.5% FS. This instrument has two modes of equal strain control and equal stress control. The specifications of the specimens included two types, one being 39.1 mm in diameter and 80 mm in height, the other 61.8 mm in diameter and 125 mm in height. In this study, it was assumed that the stress and strain would be positive under compression conditions. The stress level for the conventional triaxial creep test is expressed as *q* = *σ*_1_ - *σ*_3_, which is also called deviatoric stress. Before the creep test of the remoulded loess, the fixed confining pressure was set at 100kPa to carry out the triaxial consolidation undrained shear tests of remoulded loess under different compactness levels. Next, under the same confining pressure, indoor creep tests on different compacted remoulded loess samples were conducted under different axial deviatoric stress levels. In the initial loading, the confining pressure was applied to the designated value for 120 s. For the test, the specimens were first consolidated for 24 h at a confining pressure. After the specimens were consolidated, the axial deviatoric stress was loaded step by step according to stress levels of *R* = 20, 40, 60, 80, 90, 95, and 98% (*R* denotes the ratio of the deviatoric stress to the ultimate stress), while the axial pressure of each load on the specimens was kept constant with an accuracy of ±5 kPa for 12 h; the specific loading method for each sample is shown in Table 2. The axial loads were added stepwise until the specimens showed accelerated creep deformation. During the tests, data were automatically collected by an external strain gauge for plotting full stress-strain-time relation curves.

**Table 2:** Stress loading method (under a confining pressure of 100kPa).

Deviatoric stress level	Deviatoric stress *q* (kPa) / *R*			
	0.84	0.89	0.95	0.99
First level	75 / 0.2	99 / 0.2	122 / 0.2	133 / 0.2
Second level	150 / 0.4	198 / 0.4	244 / 0.4	267 / 0.4
Third level	225 / 0.6	297 / 0.6	366 / 0.6	400 / 0.6
Fourth level	300 / 0.8	396 / 0.8	488 / 0.8	534 / 0.8
Fifth level	337 / 0.9	446 / 0.9	550 / 0.9	600 / 0.9
Sixth level	356 / 0.95	none	none	655 / 0.95

Note: *R* denotes the ratio of deviatoric stress to ultimate stress.

## 3 Results and analyses

### 3.1 Stress-strain characteristics of remoulded loess under different levels of compactness

**Fig 4:**
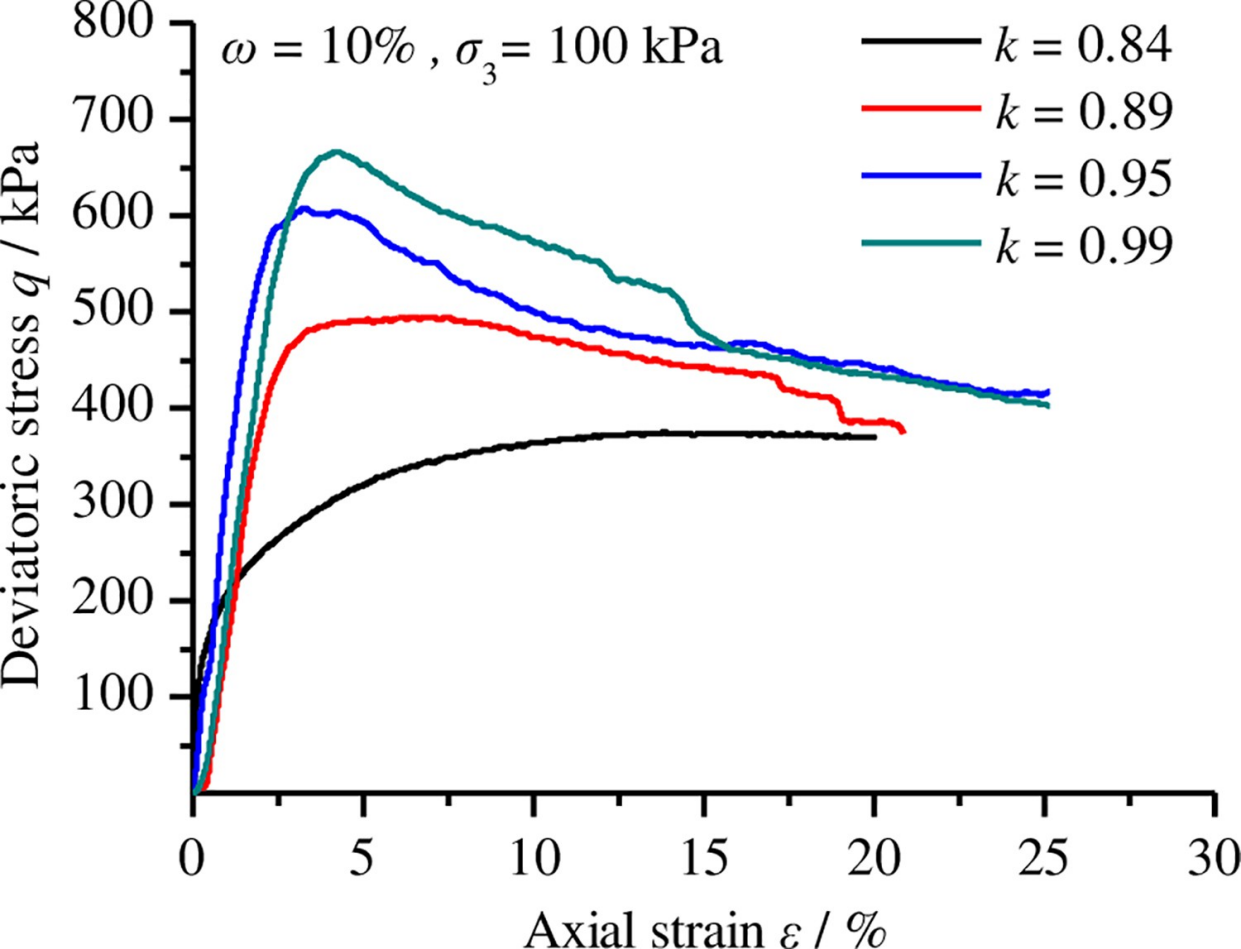
Stress-strain curves of remoulded loess with different levels of compactness based on triaxial compression test data.

Fig 4 shows the stress-strain curves obtained from remoulded loess triaxial compression tests. From the figure, we observed that (1) when the compactness is 0.84, the stress-strain curve of the remoulded loess shows strain-hardening characteristics. The axial strain increases monotonically with the increase in deviatoric stress and the shape of the curve is nearly hyperbolic. (2) When the levels of compactness are 0.89, 0.95 and 0.99, the stress-strain curves of the remoulded loess belong to the strain-softening type. In the early stages, the internal pore of the remoulded loess was compressed by the axial pressure and, thus, the growth trend of the stress-strain curves was concave. With the increase in axial stress, after the internal pore closed, the stress-strain curves grew nearly linearly, indicating the remoulded loess samples were at the elastic deformation stage until they reached the yield strength. With the axial pressure exceeding the ultimate stress, the specimen gradually expanded and started to crack. Meanwhile, the axial deviatoric stress decreased with the strain in a nearly linear way, indicating the occurrence of remoulded loess fracture. (3) The compactness affected the yield and ultimate stress. When the confining pressure was constant, the yield and ultimate stress of the remoulded loess increased as the compactness increased.

### 3.2 Creep characteristics of remoulded loess with different levels of compactness

**Fig 5:**
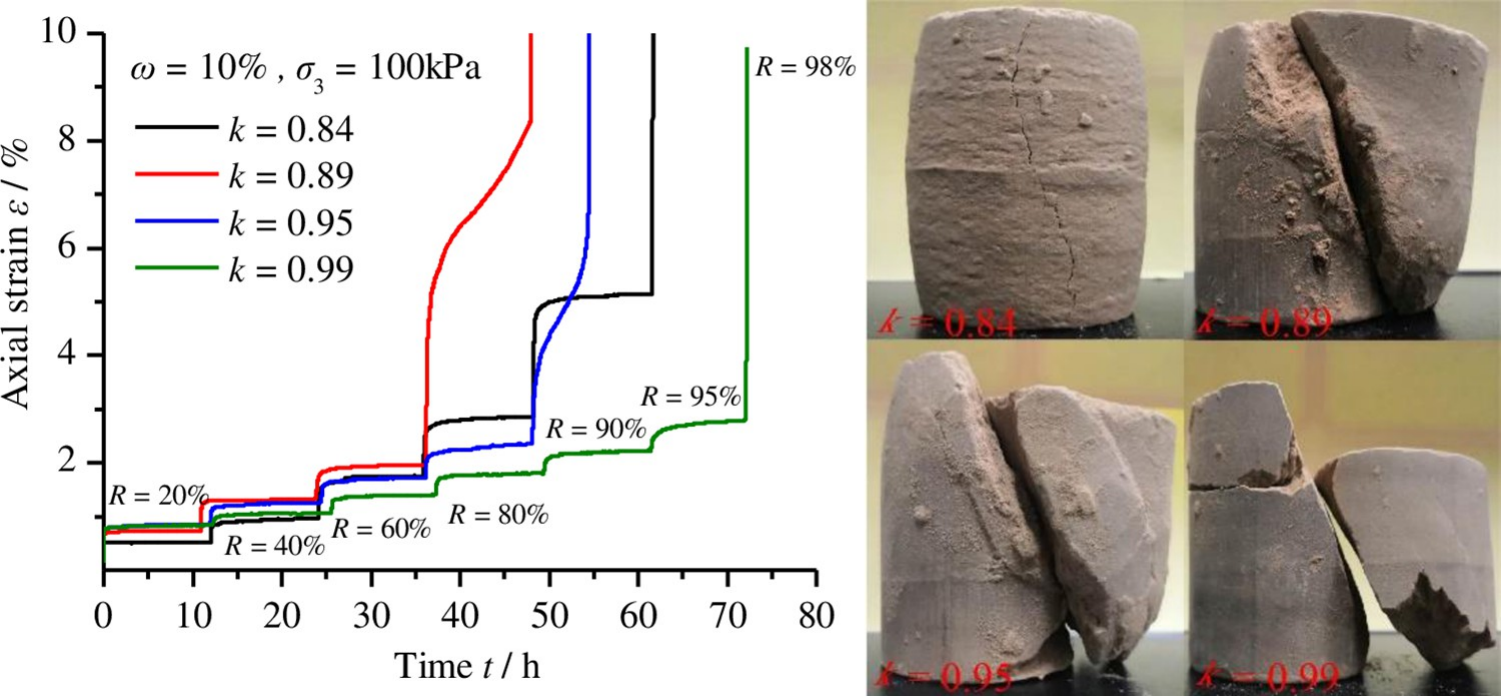
Creep curves and specimens. (a) Relationship curves of axial strain over time for remoulded loess under various levels of compactness; (b) specimens after the creep test.

**Fig 6:**
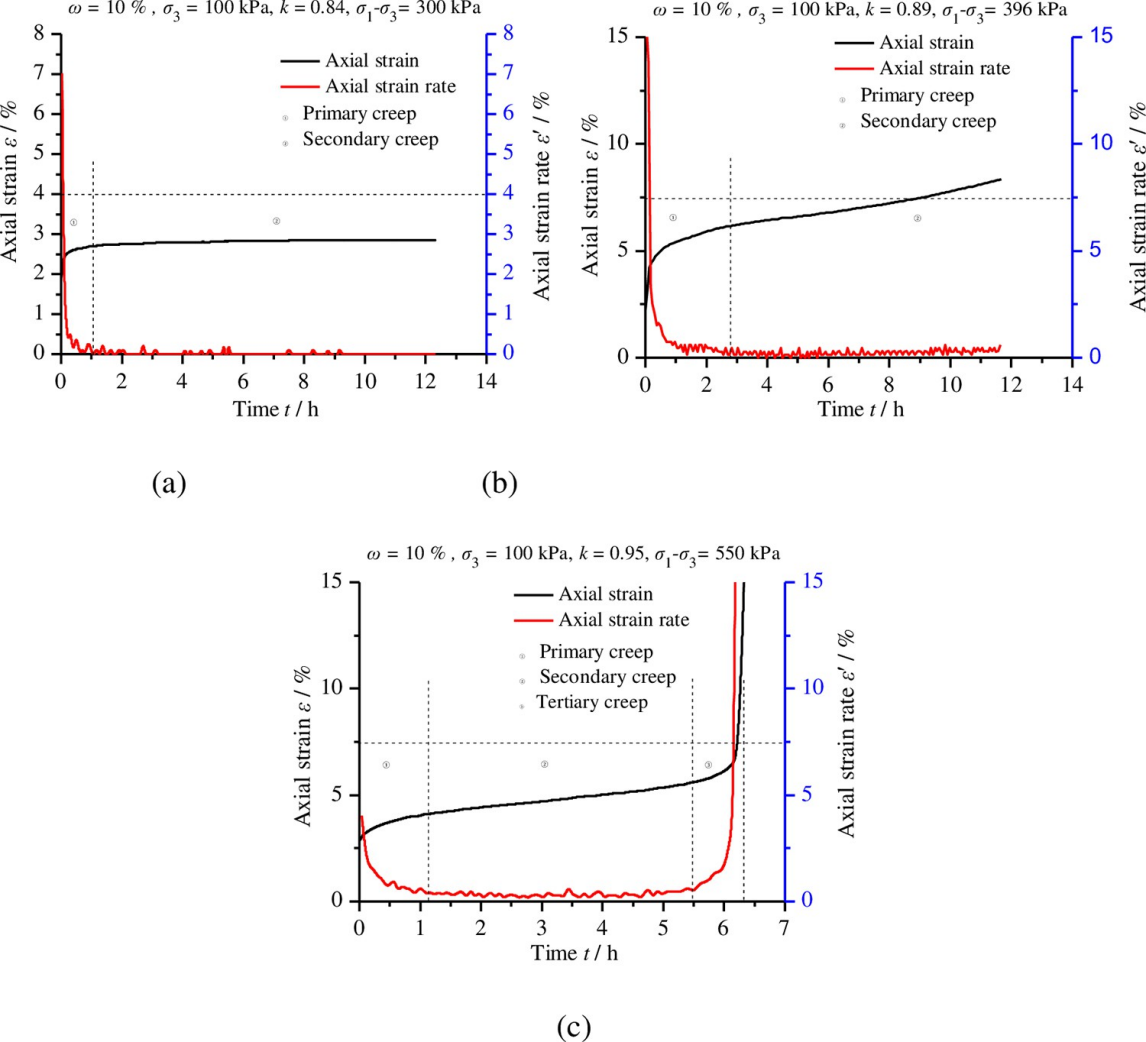
Relationship curves of axial strain and strain rate over time for remoulded loess under different deviatoric stress levels. (a) Compactness of 0.84 and deviatoric stress of 300 kPa; (b) compactness of 0.89 and deviatoric stress of 396 kPa; (c) compactness of 0.95 and deviatoric stress of 550 kPa.

Using the creep test data, the curves of the axial strain versus time at various levels of compactness were plotted, as shown in Fig 5; Fig 6 shows the partially typical relation curves of axial strain rate over time under different deviatoric stress levels. As observed in Figs 5 and 6, (1) the whole deformation process can be divided into two parts: instantaneous deformation and creep deformation. When the deviatoric stress was applied to the samples, instantaneous deformation occurred and then creep deformation developed over time. (2) Under partial deviatoric stress, we can observe three typical stages of material creep: the primary creep stage, secondary creep stage and tertiary creep stage. (3) The four samples, at each step of deviatoric stress, experienced an obvious primary creep stage when the strain rate gradually decreased. The duration of this stage was usually short and, under different deviatoric stress, the strain rate decreased to a constant value greater than or equal to zero. (4) Under some low deviatoric stress, the strain rate of the second creep stage tended to be zero under low deviatoric stress and, thus, creep strain did not accumulate with time (Fig 6(A)); however, under some high deviatoric stress, the strain rate of the second creep stage was greater than zero and constant, with the creep strain increasing linearly with time (Fig 6(B)). (5) Under some higher deviatoric stress, the strain rate of the tertiary creep stage increased rapidly with time (Fig 6(C)), the rapid accumulation of creep strain leading to the failure of the specimen. (6) Under low deviatoric stress and finite creep time, only primary creep and secondary creep occurred (Fig 6(A) and 6(B)), and the secondary creep stage lasted for a longer duration; when the deviatoric stress exceeded the yield stress, after the former two stages, the tertiary creep stage eventually occurred as time went on, leading to the occurrence of creep failure (Fig 6(C)). (7) The axial compression and radial expansion deformation of samples with low levels of compactness were more obvious than those with high levels of compactness (Fig 5(B)). (8) With the increase in compactness, the failure surface of the sample became smoother and straighter; these results indicated that the remoulded loess became hard and brittle after compaction.

### 3.3 The regularity of instantaneous strain, creep strain, total accumulated strain and initial shear modulus

**Fig 7:**
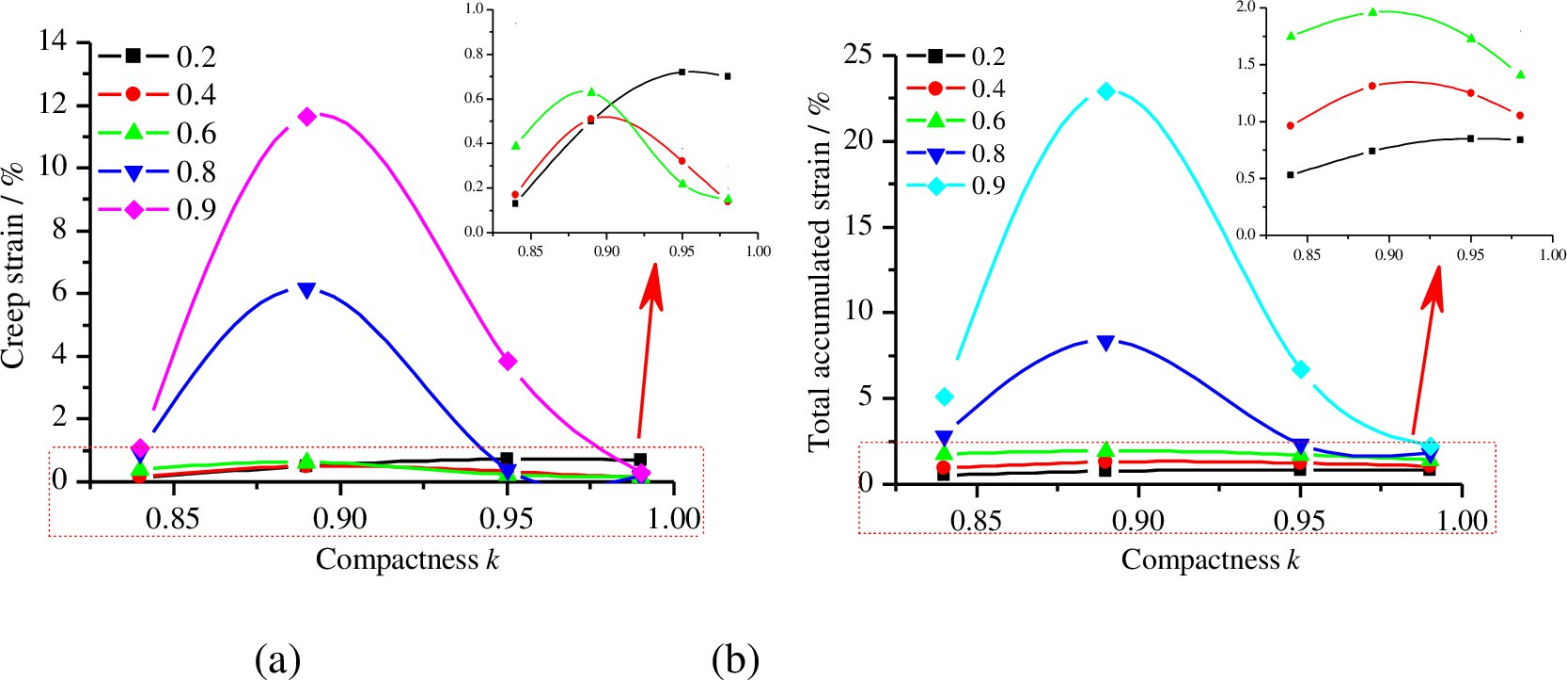
The relationship of creep strain and total accumulated strain with compactness. (a) Creep strain under different levels of compactness; (b) total accumulated strain under different levels of compactness.

**Fig 8:**
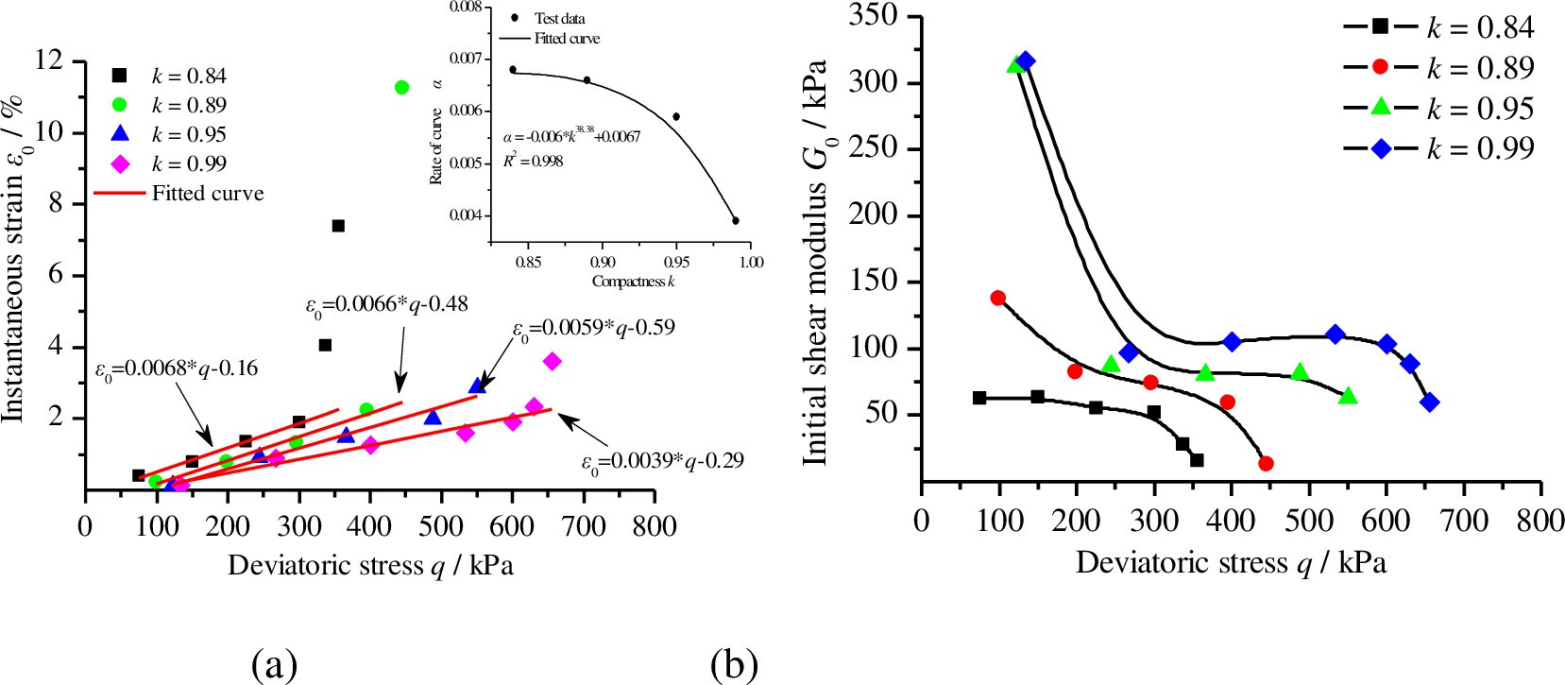
The relationship of instantaneous strain and initial shear modulus with deviatoric stress. (a) Instantaneous strain under different levels of compactness; (b) initial shear modulus under different levels of compactness.

Based on Fig 5, Boltzmann superposition processing was used to calculate the instantaneous strain, creep strain, total accumulated strain and initial shear modulus of remoulded loess under various levels of compactness, as shown in Table 3. The initial shear modulus was calculated according to Eq (1). Fig 7 shows the relationship between creep strain, total accumulated strain and compactness under different deviatoric stress ratios, respectively. Fig 8 shows the relationship between instantaneous strain, initial shear modulus and deviatoric stress under different levels of compactness, respectively.

$$\varepsilon_{0}=\frac{q}{3G_{0}},$$ (1)

where *ε*_0_ is the instantaneous strain and *G*_0_ is the initial shear modulus.

**Table 3:** The strain values of the remoulded loess under various levels of compactness.

Compactness *k*	Deviatoric stress *q* (kPa)	Deviatoric stress ratio *R*	Instantaneous strain *ε*_0_ (%)	Creep strain *ε*_c_ (%)	Total accumulated strain *ε* (%)	Initial shear modulus *G*_0_ (kPa)
0.84	75	0.2	0.40	0.13	0.53	62.5
	150	0.4	0.79	0.17	0.96	63.29
	225	0.6	1.36	0.39	1.75	55.15
	300	0.8	1.91	0.94	2.85	52.36
	337	0.9	4.05	1.09	5.14	27.74
	356	0.95	7.38	4.11	11.49	16.08
0.89	99	0.2	0.24	0.50	0.74	137.5
	198	0.4	0.80	0.51	1.31	82.5
	297	0.6	1.33	0.63	1.96	74.44
	396	0.8	2.23	6.19	8.39	59.19
	446	0.9	11.26	11.65	22.91	13.20
0.95	122	0.2	0.13	0.72	0.85	312.82
	244	0.4	0.93	0.32	1.25	87.46
	366	0.6	1.51	0.22	1.73	80.79
	488	0.8	2.00	0.38	2.38	81.33
	550	0.9	2.88	3.86	6.74	63.66
0.99	133	0.2	0.14	0.70	0.84	316.67
	267	0.4	0.91	0.14	1.05	97.80
	400	0.6	1.26	0.15	1.41	105.82
	534	0.8	1.60	0.20	1.80	111.25
	600	0.9	1.92	0.30	2.22	104.17
	630	0.95	2.36	0.43	2.79	88.98
	655	0.98	3.63	11.97	15.6	60.15

From Table 3, Figs 7 and 8, it can be observed that (1) both instantaneous strain and total accumulated strain increase with an increase in the deviatoric stress ratio and the creep strain has a similar rule when the compactness is 0.84 and 0.89. (2) Under the same deviatoric stress ratio, the creep strain and total accumulated strain of remoulded loess first increase and then decrease with the increase in compactness. (3) The initial shear modulus of the remoulded loess decreases with the increase in deviatoric stress but, at the same deviatoric stress, its initial shear modulus increases with the increase in compactness. (4) The instantaneous strain appears to change linearly with the deviatoric stress at a given compactness and, with an increase in compactness, the slopes of the fitting curves of instantaneous strain and deviatoric stress decrease accordingly. (5) The relationship between the fitting curve slope *α* and compactness can be expressed as

$$\alpha =-0.006*k^{38.38}+0.0067.$$ (2)

### 3.4 Long–term strength

**Fig 9:**
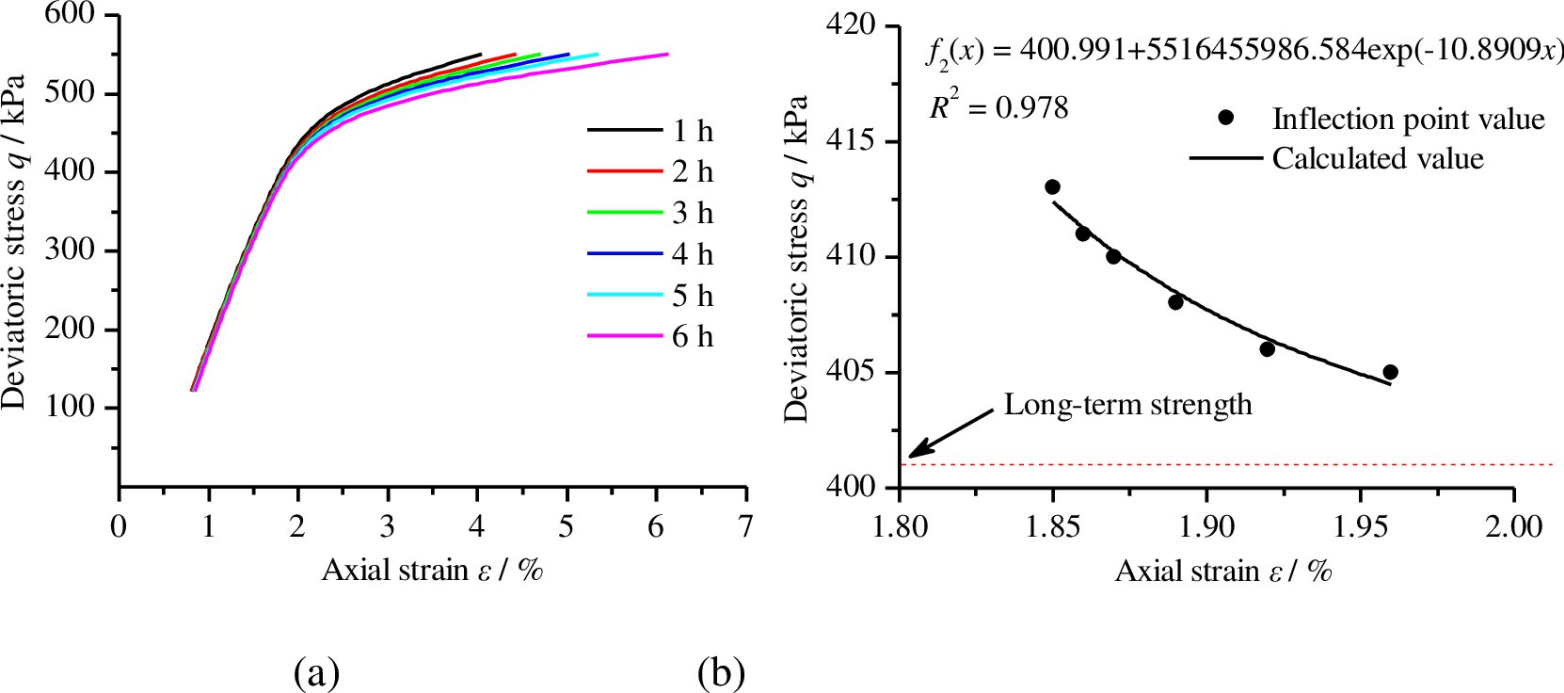
The long-term strength of remoulded loess is determined by isochronous stress-strain curves. (a) Isochronous stress-strain curves of the sample under compactness of 0.95; (b) the long-term strength was obtained by fitting the inflection point of the isochronous stress-strain curves.

Generally, when the external load on a material is less than a certain stress value, the creep deformation of the soil will gradually converge over time. However, when the external load is greater than the stress value, the creep deformation of the soil will continue to increase over time and eventually destroy it. The critical value of this stress level can be called the long–term strength of the geomaterials. The long-term strength is a key mechanical parameter to evaluate the stability and safety of materials as part of geotechnical engineering. In engineering practice, the most common method to determine the long-term strength of a material is the isochronous curve method. Isochronous stress–strain curves represent the relationship between creep strain and stress over equal time periods among a cluster of creep curves at different stress levels. It is generally believed that the long–term strength of samples corresponds with the stress at the inflection point of the isochronous curves. However, there is a high amount of error when using visual observation to determine the inflection point directly from the isochronous stress–strain curves. Therefore, we use the mathematical derivation method to determine the inflection point of the isochronous stress–strain curves [28]. Taking the isochronous stress–strain curves of the sample with a compactness of 0.95 plot in Fig 9(A) as an example, the function in Eq (3) was used to fit the isochronous stress-strain curves and the second derivative of the obtained fitting function was taken. After this, the second derivative was set equal to zero to obtain the inflection points of the isochronous stress-strain curves. Finally, the function in Eq (4) was applied to fit the inflection point of the curves so the stress value corresponding to the horizontal asymptote of the selection function *f*_2_(*x*) gives the long-term strength.


$$f_{1}(x)=ax^{3}+bx^{2}+cx,$$ (3)

$$f_{2}(x)=d+g\exp(-hx).$$ (4)

**Fig 10:**
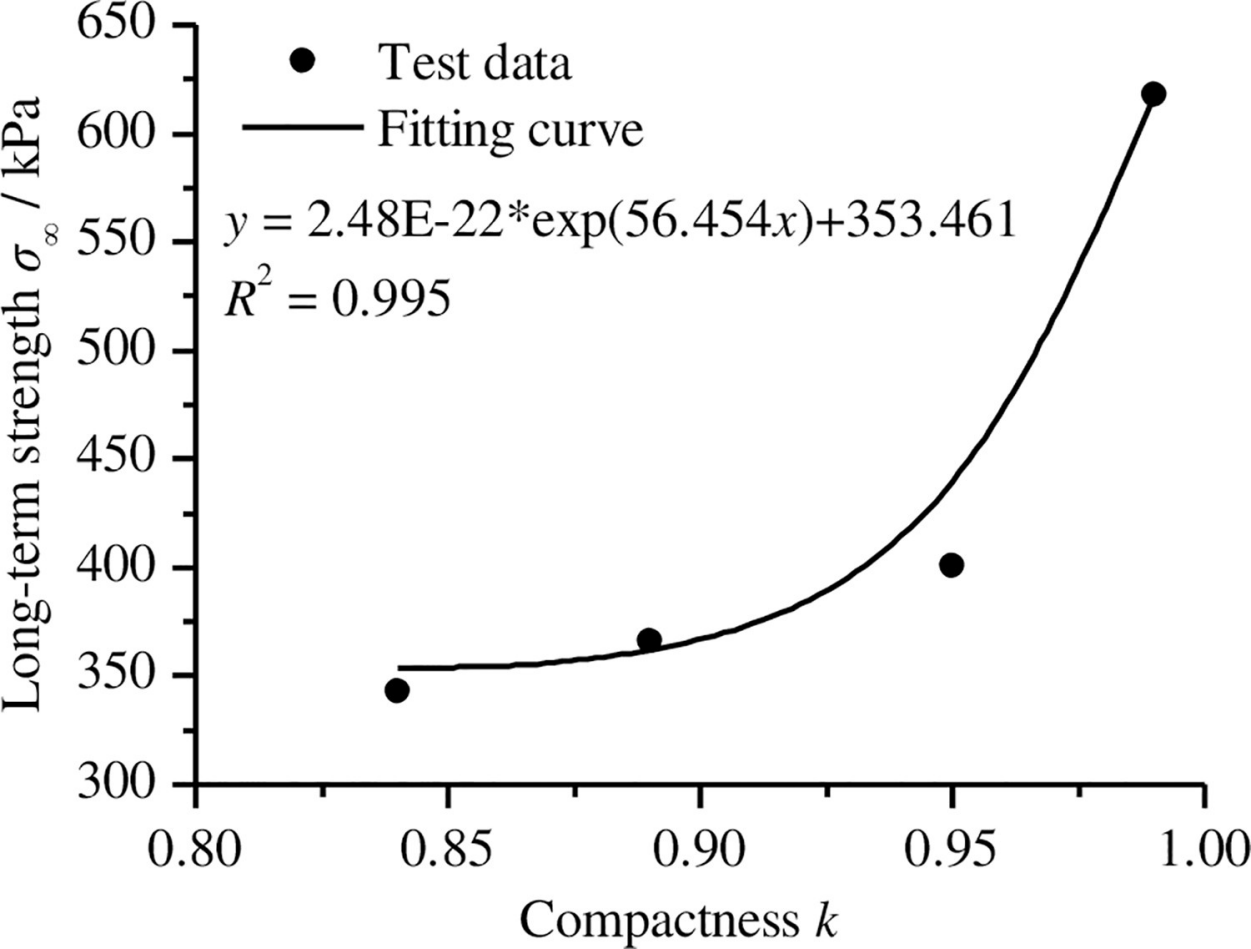
The relationship between long-term strength of remoulded loess and compactness.

As shown in Fig 9(B), the long-term strength of a sample with a compactness of 0.95 is about 400.991 kPa. According to the above method, we determined that the long-term strength of samples with compactness levels of 0.84, 0.89, and 0.99 were 343.226 kPa, 366.354 kPa, and 617.789 kPa, respectively. Fig 10 shows the relationship between compactness and the long-term strength of remoulded loess. From Fig 10, we observed that the long-term strength of remoulded loess increases significantly with an increase in compactness; the relationship between the long-term strength of remoulded loess and compactness can be expressed as

y=2.48E−22*exp(56.454x)+353.461. (5)

Where *y* is long-term strength and *x* is compactness.

## 4 Creep constitutive model of remoulded loess

### 4.1 Hardening—damage mechanism

Over several decades, considerable effort has been directed toward the study of geomaterial creep models. Various creep constitutive models for geomaterials have been proposed. Generally, they can be grouped into three categories: empirical models, component models and mechanism-based creep constitutive models. The component models have the advantage of simplicity and flexibility and are often used to describe linear creep curves. Mechanism-based creep principal structure models can better describe linear and nonlinear creep curves and the model parameters have realistic physical significance. Until now, the component creep models based on mechanical means have been paid the most attention. Hardening and damage effects accompany the creep process of geomaterials. Usually, the hardening effect starts at the beginning of loading and hardening only occurs in remoulded loess when the stress is relatively small, which is reflected in the increase of remoulded loess strength. When the stress reaches the initial threshold of creep, the remoulded loess specimens will suffer damage and deterioration due to internal dislocation and there will be obvious formation or expansion of new cracks at the macro level. Under the combined action of hardening and damage effects, the remoulded loess samples will experience creep deformation. With an increase in stress, the damage effect on remoulded loess increases, the hardening effect weakens gradually, the damage accumulates and spreads to the surroundings and the secondary cracks extend continuously. There is much less ability to resist deformation in remoulded loess; its strength weakens after the first strengthening process under the stress of higher remoulded loess samples due to the cumulative damage being too big so, eventually, crack penetration leads to failure.

### 4.2 One-dimensional creep model construction based on the hardening—damage effect

**Fig 11:**
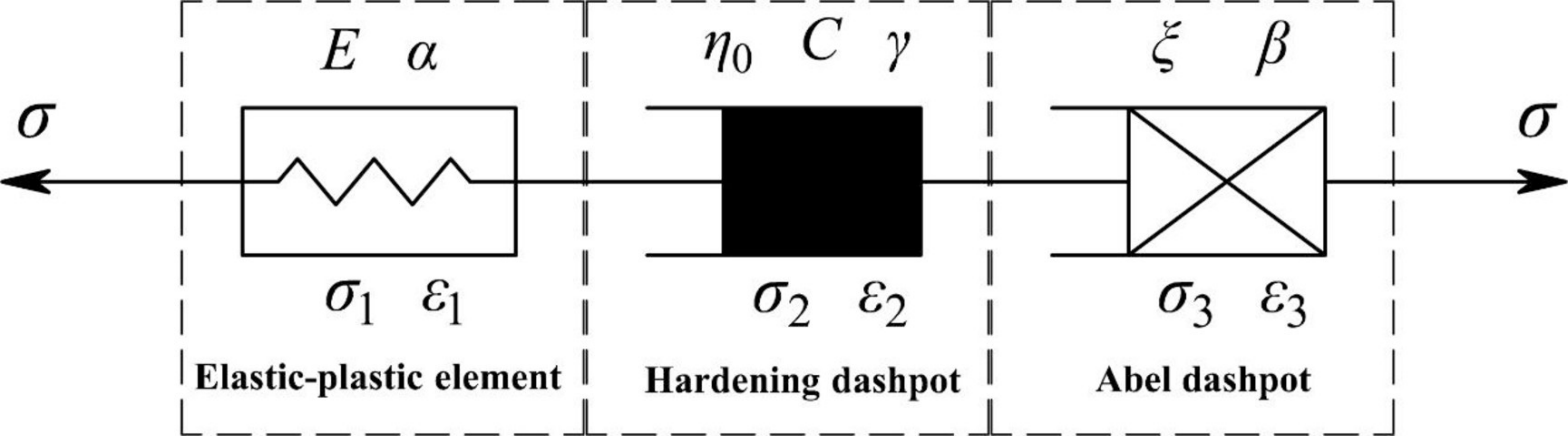
Schematic of the HD creep model.

Although there are some differences in the amount of creep deformation with remoulded loess under different levels of compactness, all the creep curves have the same basic characteristics so the creep curve can be described by the same creep constitutive model. In the test process, the stress is applied instantly and the corresponding strain curves all have instantaneous responses, indicating an independent elastic element should be included in the constitutive model. In primary creep, the strain develops over time and shows a significant correlation with time, indicating there are viscous elements in the model. Based on the analysis mentioned above, a new model which could reflect hardening-damage effects on remoulded loess during the whole process of the creep test is proposed here. The proposed model consists of an elastic-plastic element in a series with a hardening dashpot and an Abel dashpot, as shown in Fig 11, referred to as the HD creep model.

As shown in Fig 8, the elastic-plastic element based on the damage rate change was proposed by Cao et al. [29]. Based on the damage variable proposed by Kachanow [30], considering the critical stress (*σ*_s_) of the geotechnical material during creep, a new damage variable that was improved by Cao et al. [29]. It can be written as

$$D=\{\begin{array}{ll} 0 & \sigma <\sigma_{S}\\ 1-{\left(1-\frac{t}{t_{F}}\right)}^{\alpha } & \sigma \geq \sigma_{S} \end{array},$$ (6)

where *t*_F_ is the time from initial deformation to final failure and *α* is the material constant. Substituting Eq (6) into the creep constitutive relation of the spring element, the creep equation of the elastic-plastic element can be expressed as [29]

$$\varepsilon =\{\begin{array}{ll} \frac{\sigma }{E} & \sigma <\sigma_{S}\\ \frac{\sigma }{E}{\left(1-\frac{t}{tF}\right)}^{-\alpha } & \sigma \geq \sigma_{S} \end{array}.$$ (7)

Hardening dashpot is an improved element which reflects the hardening effect on soil during creep by introducing the hardening function based on the traditional dashpot creep model. The hardening function is introduced as follows

$$H=Ct^{1-\gamma },$$ (8)

where *C* and *γ* are the material constant. Substituting Eq (8) into the creep constitutive relation of dashpot, the constitutive relation of the hardening dashpot can be expressed as

$$\overset{•}{\varepsilon }=\frac{\sigma }{\eta_{0}H}=\frac{\sigma }{\eta_{0}Ct^{1-\gamma }},$$ (9)

where *η*_0_ is initial viscosity. After Solving Eq (9) by integration, the creep equation of the hardening dashpot can be expressed as

$$\varepsilon =\frac{\sigma }{\eta_{0}C\gamma }t^{\gamma }.$$ (10)

Abel dashpot is a general damping element; its constitutive relation can be expressed as

$$\sigma =\xi \frac{d^{\beta }\varepsilon }{dt^{\beta }}\;0\leq \beta \leq 1.$$ (11)

Eq (11) is known as the Scott-Blair (1947) fractional element. In Eq (11), *σ* and *ε* are the stress and strain, respectively; *ξ* is the viscosity coefficient of the material, with units in [stress·time] ^*β*^; *t* is time; and *β* is the fractional order, with 0 ≤ *β* ≤ 1. Eq (11) will degrade to demonstrate the constitutive relationship of a spring when *β* = 0 and a dashpot when *β* = 1. However, when 0 < *β* < 1, the fractional element can exhibit characteristics of both a spring and a dashpot. If considering Eq (11) in terms of its physical interpretation, it can be used to characterize the mechanical properties of the geotechnical materials between an ideal solid and an ideal fluid during creep. Thus, if the stress *σ* is a constant, the fractional element will describe the change in creep behaviour of geotechnical materials. The creep equation for materials can be obtained by employing the Riemann-Liouville fractional calculus theory as

$$\varepsilon =\frac{\sigma }{\xi }•\frac{t^{\beta }}{\Gamma (1+\beta )}\;0<\beta <1,$$ (12)

where Γ(x) is the gamma function defined as

Γ(x)=∫0∞e−ttx−1dt(Re(x)>0). (13)

Based on the structural connection characteristics of the HD creep model, as shown in Fig 8, it can be known that

$$\left\{ \begin{array}{l} \sigma =\sigma_{1}=\sigma_{2}=\sigma_{3}\\ \varepsilon =\varepsilon_{1}+\varepsilon_{2}+\varepsilon_{3} \end{array},\right.$$ (14)

Thus, the creep constitutive equations of the HD creep model in one dimension can be written as follows:

- when *σ* = *σ*_0_, and *σ*_0_ ≤ *σ*_s_, the HD creep equation is
$$\varepsilon (t)=\frac{\sigma_{0}}{E}+\frac{\sigma_{0}}{\eta_{0}C\gamma }t^{\gamma }+\frac{\sigma_{0}}{\xi }•\frac{t^{\beta }}{\Gamma (1+\beta )}\;0<\beta <1,$$ (15)
- when *σ* = *σ*_0_, and *σ*_0_ > *σ*_s_, the HD creep equation is
$$\varepsilon (t)=\frac{\sigma_{0}}{E{\left(1-\frac{t}{t_{F}}\right)}^{\alpha }}+\frac{\sigma_{0}}{\eta_{0}C\gamma }t^{\gamma }+\frac{\sigma_{0}}{\xi }•\frac{t^{\beta }}{\Gamma (1+\beta )}\;0<\beta <1.$$ (16)

### 4.3 Three-dimensional creep constitutive equations for the HD model

The triaxial compression creep stress state is *σ*_2_ = *σ*_3_ and constant; all levels of load *σ*_1_ after loading are constant. Therefore, in the 3D stress state, the total strain of the HD creep model can be expressed as tensor

$$\varepsilon_{ij}(t)=\varepsilon_{ij}{}^{1}+\varepsilon_{ij}{}^{2}+\varepsilon_{ij}{}^{3},$$ (17)

where *ε*_*ij*_(*t*) is the total strain of the HD creep model, *ε*_*ij*_^1^ is the strain of the elastic-plastic element, *ε*_*ij*_^2^ is the strain of the hardening dashpot, and *ε*_*ij*_^3^ is strain of the Able dashpot.

From the generalised Hooke’s law, the three-dimensional constitutive relation of elastomer is as follows:

$$\left\{ \begin{array}{l} e_{ij}=\frac{S_{ij}}{2G}\\ \varepsilon_{m}=\frac{\sigma_{m}}{3K} \end{array},\right.$$ (18)

where *S*_*ij*_ and *σ*_*m*_ are the partial stress tensor and spherical stress tensor; *e*_*ij*_ and *ε*_*m*_ are the partial strain tensor and spherical strain tensor; and *G* and *K* are the shear modulus and bulk modulus. Therefore, the strain of the elastomer can be written as

$$\varepsilon_{ij}=\frac{S_{ij}}{2G}+\frac{\sigma_{m}}{3K}\delta_{ij},$$ (19)

where *δ*_*ij*_ is Kronecker’s sign. Based on this transformation, the strain of the elastic-plastic element can be expressed as

$$\varepsilon_{ij}{}^{1}=\{\begin{array}{ll} \frac{S_{ij}}{2G}+\frac{\sigma_{m}}{3K}\delta_{ij} & S_{ij}<\sigma_{s}\\ \frac{S_{ij}}{2G{\left(1-\frac{t}{t_{F}}\right)}^{\alpha }}+\frac{\sigma_{m}}{3K}\delta_{ij} & S_{ij}\geq \sigma_{s} \end{array}.$$ (20)

If the material rheology is mainly governed by shear deformation, the 3D constitutive relationship between the hardening dashpot and Abel dashpot can be expressed as

$$\left\{ \begin{array}{l} \varepsilon_{ij}{}^{2}=e_{ij}{}^{2}=\frac{S_{ij}}{2\eta_{0}C\gamma }t^{\gamma }\\ \varepsilon_{ij}{}^{3}=e_{ij}{}^{3}=\frac{S_{ij}}{2\xi }•\frac{t^{\beta }}{\Gamma (1+\beta )} \end{array},\right.$$ (21)

where *e*_*ij*_^2^ and *e*_*ij*_^3^ are the partial strain tensors of the hardening and Abel dashpots, respectively.

Based on Eqs (20) and (21), the 3D creep equation of the HD creep model can be written as

$$\left\{ \begin{array}{lll} \varepsilon_{ij}=\frac{S_{ij}}{2G}+\frac{\sigma_{m}}{3K}\delta_{ij}+\frac{S_{ij}}{2\eta_{0}C\gamma }t^{\gamma }+\frac{S_{ij}}{2\xi }•\frac{t^{\beta }}{\Gamma (1+\beta )} & S_{ij}<\sigma_{s} & 0<\beta <1\\ \varepsilon_{ij}=\frac{S_{ij}}{2G^{*}}+\frac{\sigma_{m}}{3K}\delta_{ij}+\frac{S_{ij}}{2\eta_{0}C\gamma }t^{\gamma }+\frac{S_{ij}}{2\xi }•\frac{t^{\beta }}{\Gamma (1+\beta )} & S_{ij}\geq \sigma_{s} & 0<\beta <1 \end{array},\right.$$ (22)

where G* = *G*(1-*D*).

When the triaxial compression creep stress state is *σ*_2_ = *σ*_3_ and constant, all levels of load *σ*_1_ are constant; then

$$\left\{ \begin{array}{l} \sigma_{m}=\frac{1}{3}(\sigma_{1}+2\sigma_{3})\\ S_{11}=\sigma_{1}-\sigma_{m}=\frac{2}{3}(\sigma_{1}-\sigma_{3}) \end{array},\right.$$ (23)

Thus Eq (13) can be rewritten as

$$\{\begin{array}{l} \varepsilon_{ij}=\frac{\sigma_{1}-\sigma_{3}}{3G}+\frac{\sigma_{1}+2\sigma_{3}}{9K}+\frac{\sigma_{1}-\sigma_{3}}{3\eta_{0}C\gamma }t^{\gamma }+\frac{\sigma_{1}-\sigma_{3}}{3\xi }•\frac{t^{\beta }}{\Gamma (1+\beta )}\\ \;\sigma_{1}-\sigma_{3}<\sigma_{s}\;0<\beta <1\\ \varepsilon_{ij}=\frac{\sigma_{1}-\sigma_{3}}{3G{\left(1-\frac{t}{t_{F}}\right)}^{\alpha }}+\frac{\sigma_{1}+2\sigma_{3}}{9K}+\frac{\sigma_{1}-\sigma_{3}}{3\eta_{0}C\gamma }t^{\gamma }+\frac{\sigma_{1}-\sigma_{3}}{3\xi }•\frac{t^{\beta }}{\Gamma (1+\beta )}\\ \;\sigma_{1}-\sigma_{3}\geq \sigma_{s}\;0<\beta <1 \end{array}.$$ (24)

### 4.4 Parameter determination for the HD creep model by fitting analysis

**Fig 12:**
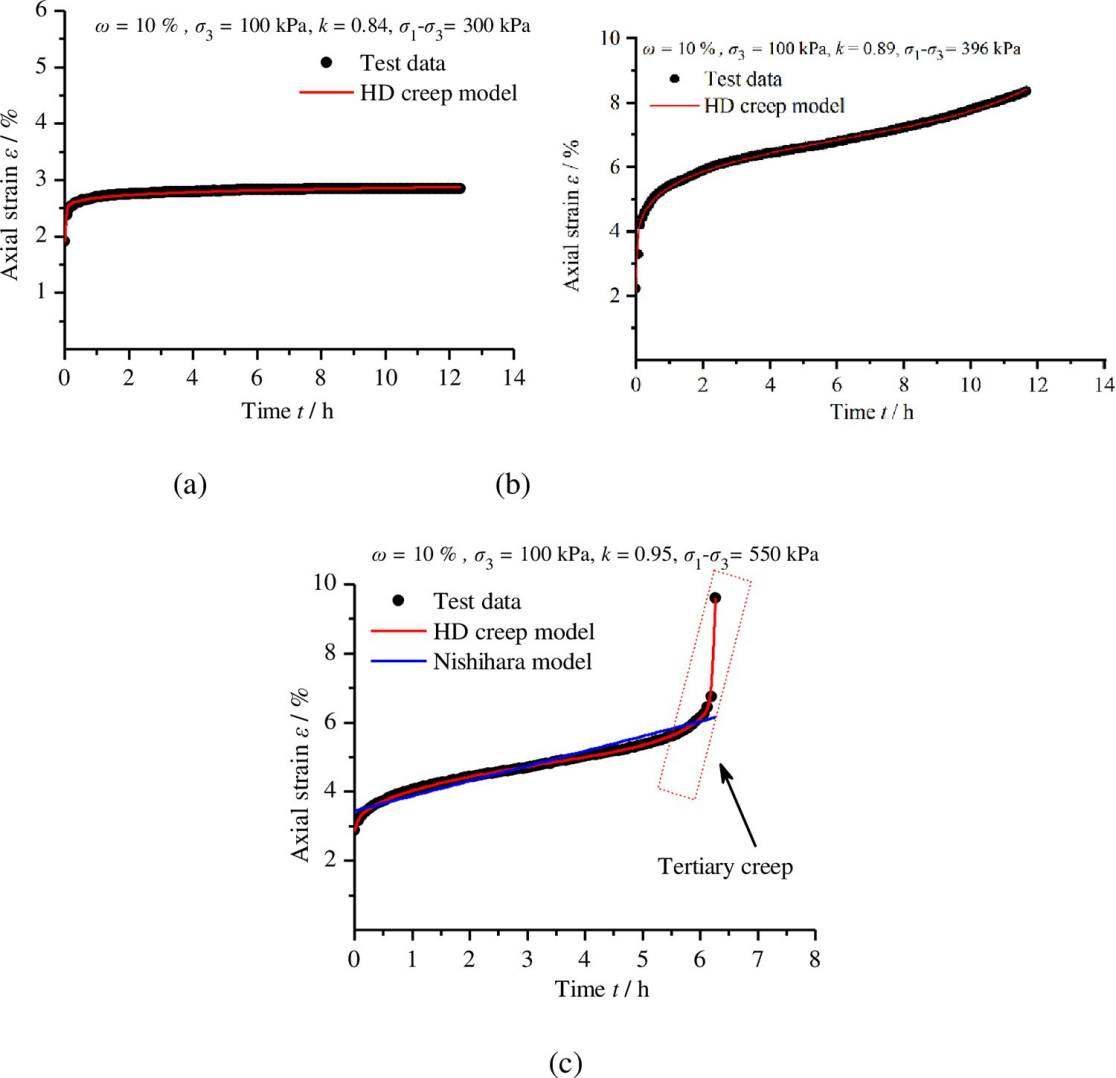
Test data of remoulded loess and fitting curves. (a) Compactness of 0.84 and deviatoric stress of 300 kPa; (b) compactness of 0.89 and deviatoric stress of 396 kPa; (c) compactness of 0.95 and deviatoric stress of 550 kPa.

The efficacy of the proposed model is dependent on its ability to adequately fit experimental data. All parameters in the HD creep model can be determined from the experimental tests using the fitting method. Using the experimental data of the remoulded loess creep in Fig 6, the parameters of the HD creep model were determined by the Levenberg–Marquardt method according to Eq (24); the results are presented in Table 4 and Fig 12. As shown in Fig 12(A), when the deviatoric stress is lower than the yield stress, the data obtained in the creep test are well fitted by Eq (24), the primary creep and secondary creep of remoulded loess can be captured by the proposed model. Fig 12(B) and 12(C) validate the proposed model in the scenario where the deviatoric stress is higher than the yield stress; as observed, the HD creep model proposed in this paper can adequately represent the creep deformation of remoulded loess. With a compactness of 0.95 and a deviatoric stress of 550 kPa, as shown in Fig 12(C), the Nishihara model was used to fit the test data and compare the results with the HD creep model. The comparisons between experimental and theoretical curves of different models suggest the HD creep model proposed in this paper can provide a precise description of full creep stages. In particular, the tertiary creep stage of remoulded loess is well represented by the proposed model.

**Table 4:** Parameters determined through fitting the analysis based on creep tests of remoulded loess.

HD creep model	*k*	*σ*_1_-*σ*_3_/kPa	*G*/kPa	*K*/kPa	*η*_0_/kPa·h	*C*	*γ*	*ξ* /kPa·h^*β*^	*β*	*α*	*σ*_s_/kPa	*t*_*F*_/h	*R* ^2^
	0.84	300	471.761	39.621	404.289	10.709	0.086	0.005	0.086		343.226		0.982
	0.89	396	13604.301	36.847	5.801	51.207	0.197	0.007	0.197	37.048	366.354	98.543	0.998
	0.95	550	270666.446	33.192	5913.381	6161.753	7.736	0.006	0.423	1.616	400.991	6.3	0.999
Nishihara model	*k*	*σ*_1_-*σ*_3_/kPa	*G*_0_/kPa	*K*/kPa	*G*_*k*_/kPa	*η*_*k*_/kPa·h	*η*_*s*_/kPa·h	*σ*_s_/kPa					*R* ^2^
	0.95	550	80.301	71.612	0.155	28047.865	47.019	490					0.797

### 4.5 Sensitivity analysis of parameters

It is obvious that the time-dependent strain of remoulded loess depends on many parameters, as shown in Eq (24); among these, the exponent *α*, and *γ*, and the fractional derivative order *β* are the most important. As we know, the secondary creep and tertiary creep stages of remoulded loess are very important for deformation prediction in loess engineering. To better understand how changes in the HD creep model parameters affect the secondary creep and tertiary creep stages, the HD creep model parameter obtained from Fig 12(C) is taken as an example to analyse the sensitivity of the three parameters (*α*, *γ*, and *β*).

**Fig 13:**
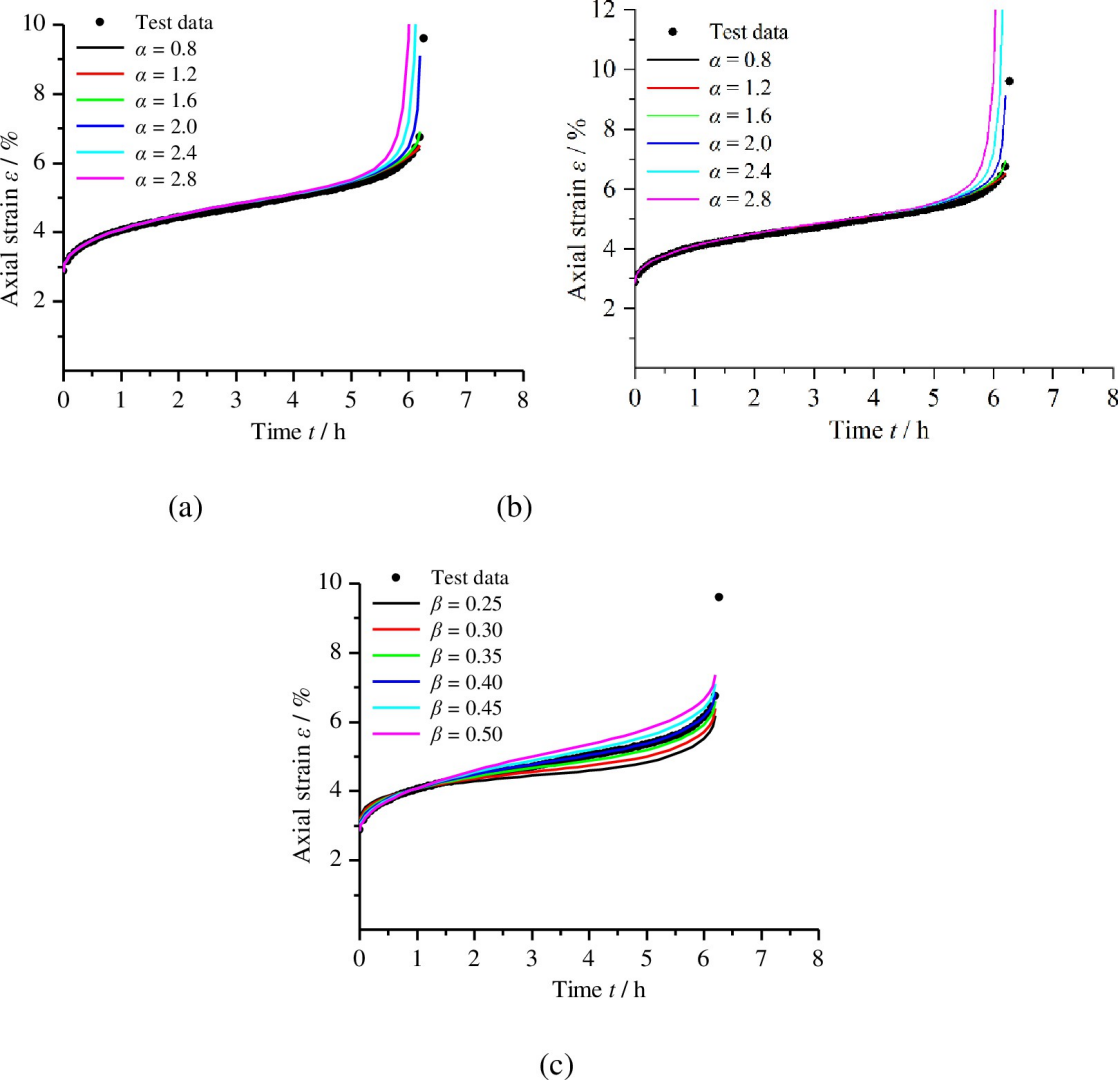
Sensitivity of the creep strain to the parameters. (a) The effect of *α*; (b) the effect of *γ*; (c) the effect of *β*.

Letting the nonstationary coefficient *α* change from 0.8 to 2.8 with an interval of 0.4, with other parameters fixed as constants, we obtained a series of curves for various *α* levels, as shown in Fig 13(A). Clearly, at the tertiary creep stage, the creep strain and strain rate strongly depended upon *α*, with large *α* values resulting in large creep strain rates, indicating that *α* is very flexible in describing the creep model.

Letting the nonlinear coefficient *γ* change from 5 to 10 with an interval of 1, with other parameters being constant, we obtained a series of curves, as shown in Fig 13(B). The figure indicates that the time period of the secondary creep stage is shorter with an increase in *γ* level, and, when *γ* increases, the creep strain rate of the tertiary creep stage becomes smaller but the tertiary creep stage occurs faster.

Considering the effects of the fractional derivative order *β*, when other parameters were held constant, a series of curves were obtained by changing *β* from 0.25 to 0.5 with an interval of 0.05, as shown in Fig 13(C). At the secondary creep and tertiary creep stage, the creep strain and strain rate increased with the increase in fractional derivative order *β*.

## 5 Conclusions

The conventional triaxial compression and triaxial creep of remoulded Q_2_ loess under different levels of compactness were examined in this study. Using the results of this study, several conclusions can be drawn.

Triaxial conventional compression tests were conducted under varying levels of compactness in the study area. The test results showed that the remoulded loess becomes harder and has higher mechanical strength with an increase in compactness. In addition, the triaxial creep tests under different levels of compactness were performed. The results showed that remoulded loess experienced three stages but that different deformation characteristics appeared under different deviatoric stress values. With an increase in compactness, the instantaneous elasticity and long-term strength of the remoulded loess improved.

A hardening dashpot was introduced. Based on the hardening-damage mechanism, in combination with an elastic-plastic element, a hardening dashpot and an Abel dashpot, the HD creep model was proposed to describe the curve of remoulded loess creep tests. The 3D constitutive equations of the HD creep model were developed and used to fit remoulded loess creep curves under various deviatoric stress levels. The results showed that the theoretical curves of the HD creep model were in higher agreement with the triaxial creep test data, especially for the tertiary creep stage, compared with the Nishihara model.

The sensitivity of the HD creep model parameters was analysed and indicated that the parameters of *α*, *γ*, and *β* significantly affect the morphological changes and various stage characteristics of the creep curve. From another point of view, the creep effect of the remoulded loess under different environments can be simulated by changing the above parameters. Thus, these parameters are highly useful in controlling the HD creep model and should be studied further.

## Supporting information

S1 File (ZIP)
