# Supplementary material for: Experimental investigation of the creep behaviour of remoulded loess under different levels of compactness
Source: PLoS One. 2022 Jan 24;17(1):e0262456. doi: 10.1371/journal.pone.0262456 (PMC8786181; doi:10.1371/journal.pone.0262456)
Supplement: S1 File — (ZIP) [file pone.0262456.s001.zip › polish.pdf]

NATIVE English Editing

https: www.nativeee.com

Address: 18 East Jiuxianqiao Road,

Chaoyang District, Beijing, China

Phone: +861064125081

26th September, 2021

STATEMENT OF EDITING

This is to certify that the following document has been checked and corrected for proper English language, grammar, punctuation, spelling, and overall style by one or more of the highly-qualified, native English-speaking editors at Native English Editing.

Native English Editing provides editing and proofreading of scientific manuscripts for submission to peer-reviewed journals.

Manuscript title: Experimental investigation of the creep behaviour of remoulded loess under different levels of compactness

Date Issued: 26th September, 2021

Certificate Verification Key: 2021092357519710

Yours truly,

Graeme Brown

Co-owner Native English Editing

Email: publish@nativeee.com

Contact information of Beijing sales department in China:

Address: 18 East Jiuxianqiao Road, Chaoyang District, Beijing, China

Phone: +861064125081

Fax: +861064125081

Contact information of Australian editorial department:

Address: 42D Melrose Street, Parkdale Vic 3195, Australia

Phone: +61417560758
